# A New Polymer-Based Mechanical Metamaterial with Tailorable Large Negative Poisson’s Ratios

**DOI:** 10.3390/polym12071492

**Published:** 2020-07-03

**Authors:** Shanshi Gao, Weidong Liu, Liangchi Zhang, Asit Kumar Gain

**Affiliations:** 1 Laboratory for Precision and Nano Processing Technologies, School of Mechanical and Manufacturing Engineering, University of New South Wales, NSW 2052 Sydney, Australia; shanshi.gao@student.unsw.edu.au (S.G.); weidong.liu.unsw@gmail.com (W.L.); a.gain@unsw.edu.au (A.K.G.); 2 Department of Mechanics and Aerospace Engineering, Southern University of Science and Technology, 518055 Shenzhen, China

**Keywords:** metamaterial, negative Poisson’s ratio, 3D printing, finite element analysis, uniaxial tension/compression

## Abstract

Mechanical metamaterials have attracted significant attention due to their programmable internal structure and extraordinary mechanical properties. However, most of them are still in their prototype stage without direct applications. This research developed an easy-to-use mechanical metamaterial with tailorable large negative Poisson’s ratios. This metamaterial was microstructural, with cylindrical-shell-based units and was manufactured by the 3D-printing technique. It was found numerically that the present metamaterial could achieve large negative Poisson’s ratios up to −1.618 under uniaxial tension and −1.657 under uniaxial compression, and the results of the following verification tests agreed with simulation findings. Moreover, stress concentration in this new metamaterial is much smaller than that in most of existing re-entrance metamaterials.

## 1. Introduction

Metamaterials are types of artificial materials with ingenious internal structure and extraordinary properties which have not been found in natural materials [1,2]. The properties of a metamaterial can be tailorable by a programed design of the internal microstructure [1,3]. Such micro-structured metamaterials were previously challenging to be manufactured using traditional processing techniques. However, the recent emergence of advanced 3D-printing techniques has removed the barriers [4,5,6].

Poisson’s ratio is one of the most important mechanical properties of materials [7]. The Poisson’s ratio of natural material is between 0 and 0.5 [8]. For a mechanical metamaterial, however, its Poisson’s ratio can be tailorable in a wide range, even to a negative value through its microstructural design. Lakes [9] reported a metamaterial with a negative Poisson’s ratio (NPR) of −0.17 using re-entrant structural cells. Mizzi et al. [10] designed a negative Poisson’s ratio metamaterial using star-shaped pores with connection beams as its microstructure. The deformation modes of the pores and beams in this metamaterial consisted of rotating mechanism and re-entrant mechanism to customize the overall very different Poisson’s ratios [11,12]. Nežerka et al. [13] proposed a modular oval-pored metamaterial with adjustable Poisson’s ratios using a puzzle-like interlocking structure [14]. Following the similar interlocking concepts, a double-negative mechanical metamaterial was designed, and achieved both negative stiffness and Poisson’s ratio [15]. Yang and Ma [16] fabricated a metamaterial with a near-zero Poisson’s ratio using a bistable arched-bridge structure. The curved-beam elements could accommodate deformation by shifting between its stable states [17,18]. A similar internal structure with arch beams was also proposed in other metamaterial designs [19,20].

Paper folding mechanisms have been utilized widely in the development of metamaterials with tailorable Poisson’s ratios. Neville et al. [21] designed morphing Kirigami mechanical metamaterials by introducing cutting pores in honeycomb structures and adjusted their Poisson’s ratio as well as fold stiffness by varying fold angles. Olympio et al. [22] reported that foldable hexagon and re-entrant structures could produce metamaterials of positive and negative Poisson’s ratios, respectively, such that the formulated combination of these two structures can bring about a metamaterial with an expected Poisson’s ratio. Based on a similar idea, Broccolo et al. [23] proposed a cellular structure for achieving zero Poisson’s ratio. However, most of these Kirigami-inspired metamaterials used straight or curved beams as the major components in their internal structure. The prior study confirmed that these network-embedded internal structures significantly reduce stiffness and strength [24]. To overcome this problem in Kirigami-inspired metamaterials, sinusoidal-shaped ribs and extra vertical ribs were added to the original hexagonal and re-entrant units [25]. Alternatively, the rhombic configuration was placed in the re-entrant honeycomb to improve in-plane stiffness while maintaining the negative Poisson’s ratio of the original re-entrant structure [26]. Combining pore-embedded unit and re-entrant structure, Li et al. [27] proposed a butterfly-like perforated 3D structure, and its soft structure could achieve a negative Poisson’s ratio by squeezing the pores inside the unit. Different stiffness ratios could be adjusted by varying the geometry parameters. Jiang et al. proposed an NPR mechanical metamaterial with circular honeycomb structures instead of recessed hexagonal honeycomb structures. In their design, semicircular structures and straight connection beams were combined into a core and periodically arranged as a 2D structure. They established the equivalent elastic modulus of this metamaterial and compared the structural equivalent elastic modulus as well as Poisson’s ratio through adjusting the radius and thickness of semicircular structures using FEA tools [28]. However, the curvature of circular structures could bring a significant influence in Poisson’s ratios, and they only considered a semicircular case. Further, they developed the equivalent elastic modulus and structural Poisson’s ratio only with the finite element method without physical experiments.

Although various Poisson’s ratio related metamaterials have been designed and manufactured, most of them have limitations in practical applications. For example, in order to get a more significant in-plate deformation, the substrate materials of star-shaped-pore metamaterials and liner pore metamaterials were made of soft polymers (*E* = 0.68 GPa) [13]. Moreover, only the flat structure of the star-shaped-pore metamaterials was involved currently, showing the monotonous deformation trend of these metamaterials under uniaxial loadings. The lack of strength would limit their promotions in engineering applications. For bistable bridge metamaterials, theoretically a large longitudinal deformation with almost zero Poisson’s ratio can be achieved, but the longitudinal stretch brought impulse into the internal structure when the arched bridges shifted its position from one stable state to another and may lead to material failure [29]. In metamaterials based on the paper folding mechanism, the deformation is achieved by the release of the folded structures, and thus thin beams are required, which would reduce structural stability. Although in some designs, reinforcing ribs can increase structural stiffness, the contradiction between large deflection and structural rigidity due to the introduction of reinforcements was not handled reasonably. Furthermore, the sharp corners in the microstructures would lead to severe stress concentration [30]. As such, the metamaterials with the Kirigami cellular structure were proposed for improving the stiffness of the metamaterials based on the paper folding mechanism under bending conditions [31,32]. However, solutions to longitudinal compression and tension are unavailable. Due to its weak structural stability under longitudinal compression, Kirigami metamaterial does not have practical applications at this stage [22,31,32]. According to the literature, Table 1 summarizes various types of Poisson’s ratio related metamaterials with their structural limitations.

Thus, it is meaningful to design NPR mechanical metamaterial for engineering applications. This metamaterial could achieve its unique deformation behavior without the limitation of materials, and the deformation process under uniaxial load is smooth and continuous. Meanwhile, the disadvantages of stress concentration in conventional paper-folding inspired structures need to be minimized.

This paper aims to develop a mechanical metamaterial with tailorable large negative Poisson’s ratios and less internal stress concentration. This metamaterial has a novel internal structure and strong 3D expansion capabilities. First, a new design of the mechanical metamaterial with negative Poisson’s ratio will be proposed on the basis of cylindrical-shell-beam units. The finite element analysis (FEA) will be carried out to predict its performance. The designed metamaterial will be manufactured using a stereolithography 3D printer then. Finally, uniaxial tension and compression tests will be conducted to evaluate the Poisson’s ratio of the metamaterial.

## 2. Methodology

### 2.1. Microstructural Design

**Figure 1:**
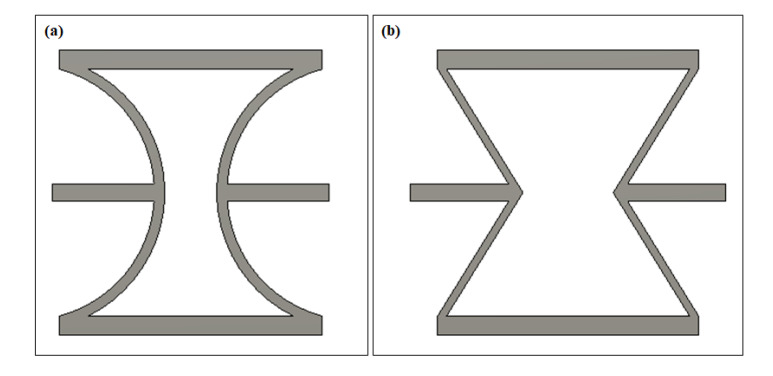
Negative Poisson’s ratio metamaterial units (**a**) cylindrical-shell (CS)-based unit and (**b**) re-entrant unit.

Figure 1 shows the proposed cylindrical-shell-based (CS-based) metamaterial unit as well as the commonly used re-entrant metamaterial unit. Our new design uses cylindrical shells to obtain continuous and controllable deformation with tailorable negative Poisson’s ratios, and the deformation mode can be changed from folding to bending. At the same time, the internal deformation distribution in an individual unit is more uniform, resulting in a significant improvement in increasing structural stability. Horizontal straight beams are added between units as connections.

**Figure 2:**
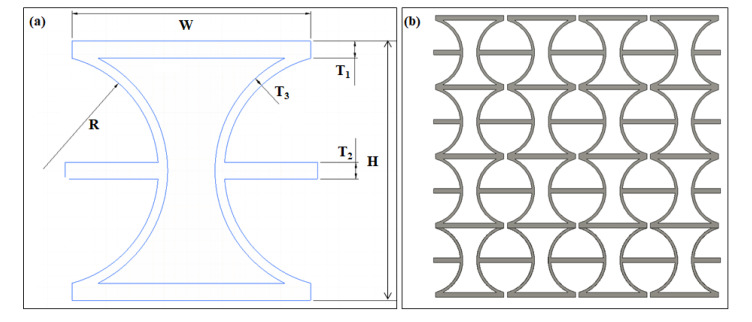
(**a**) Geometry profile of a CS-based unit and (**b**) the metamaterial structure.

Figure 2 shows the geometry profiles of the CS-based unit and the 2D metamaterial structure. The width (*W*), height (*H*), and thickness of an individual unit are the main geometry parameters. (*R*) indicates the radius of the circular rings corresponding to the cylindrical shells between two horizontal beams. These two horizontal beams are always placed on both sides of the center of the circle symmetrically. With a fixed height (*H*) in each unit, variations in *R* values make the arc between horizontal beams different, further resulting in differing curvatures of cylindrical shells. *R* is one of the core parameters in the geometry design. It could bring various deformation capacities of units. In the following sections, the performances of models with different cylindrical shell angles will be compared. Another critical parameter in geometry design is the relative thickness of different parts in an individual unit. As shown in Figure 1a, two kinds of components were assembled in an individual unit, the horizontal straight beams as connections and cylindrical shells as the deformation elements. The thickness ratio of the horizontal beams to the cylindrical shells determines the deformation trend and influences the Poisson’s ratio. In the current design (Figure 2a), the straight beams (*T_1_*) located on the top and bottom were thicker than the middle connection beams(*T_2_*), and the thickness of the cylindrical shells (*T_3_*) was lower than both straight beams. As a result, significant deformation would happen in thinner cylindrical-shell-microstructures, while the thicker straight beam possesses a higher stiffness and keeps the required rigidity of the structure under sizeable uniaxial loadings.

### 2.2. Finite Element Analysis

A commercial finite element analysis software, Ansys Workbench 19.1, was utilized to simulate the deformation of the CS-based units (unit simulation) and the 2D metamaterial structures (structure simulation).

The finite element analysis conducted in this paper was on the basis of the following assumptions.

- Geometries of the specimens are assumed to be perfect. Surface and internal defects by the manufacturing process are negligible.
- The raw material applied to this design is isotropic, the possible anisotropy of structure due to 3D printing technique is neglected.
- Fixed boundaries are applied onto the bottom surface as a simplification. Loadings are displacement-controlled and applied to the top surface of the specimen.
- There is no out-plane distortion during compression.

In the unit simulations, the Poisson’s ratio and internal stress distribution were compared between conventional re-entrant and CS-based units under uniaxial tension. The Poisson’s ratio performance of different cylindrical-shell curvatures was also studied. The primary geometry parameters, including the size of units, the thickness of beams, and the relative positions of beams were consistent for both re-entrant and CS-based units. The overall structure simulation model consisted of 4 × 10 CS-based units. Both uniaxial tensile and compressive loadings were applied to this 2D structure model. The loadings involved in this paper is displacement controlled.

The mechanical properties of the raw material (Durable resin) were set as follows: Poisson’s ratio of resin *v* = 0.35; density: 1.08 g/cm^3^ [33].

As this resin is viscoelastic [34], the definition of Young′s modulus and ultimate tensile strength could not be copied from the technical information from the company. Pre-tests have been conducted following ASTM D638 and ASTM D695 to understand the deformation behavior of this material in elastic and plastic regions [35,36]. The stress–strain curves of this resin under tension and compression were imported into Ansys Workbench 19.1 as part of the material property definition.

**Figure 3:**
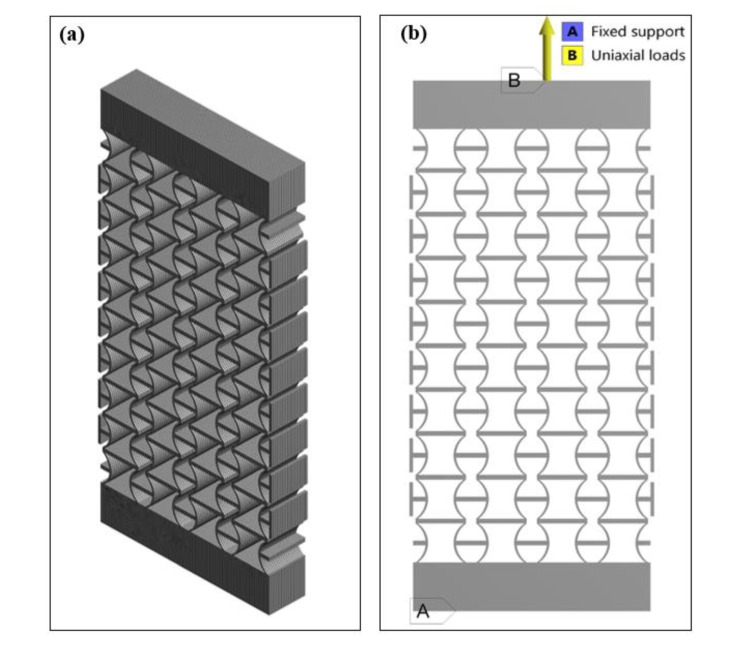
(**a**) Element division and (**b**) boundary conditions in the structure simulation.

Fixed supports were applied onto the bottom surface of models, while uniaxial displacements were applied on the top surface as boundaries. SOLID 45 elements with eight nodes and three translational degrees have been used in the simulations. Edge sizing techniques were applied in the simulation process to refine the mesh. Cylindrical shells and horizontal beams connected to them were focused as key points, lines, or areas. Different numbers of divisions and bias on such edges were adjusted to guarantee the meshing was uniform without sudden changes in adjacent grids. This model has meshed with more than 69600 elements. Grids with different sizes have been compared, and there was not a significant improvement in accuracy as a result. To increase simulation accuracy further, the large deflection option was activated. Figure 3 shows the detailed element division and boundary conditions in the structure simulation.

### 2.3. 3D-Printing and Uniaxial Tensile/Compressive Tests

A 3D printer using the stereolithography apparatus (SLA) technique was used to build the designed mechanical metamaterial [37,38,39]. Both objects and their supporting structures were 3D printed together using the same durable resin by layers. The accuracy of the objects could be controlled by the resolution of the laser beam, processing speed, and post-processing methods [38,39]. In the present study, the layer thickness during 3D-printing and touchpoint size were set as 0.1 mm and 0.5 mm, respectively. After 3D-printing, the specimens were placed in an 80 °C heating box for 20 min to stabilize the 3D-printed internal structure. In the final stage, the attached supporting structures were cut off.

Vernier calipers were used to measure the dimensions of 3D-printed specimens. Random measuring points were taken on different surfaces of samples, and the number of the measuring points for each dimension was 10. The results were summarized in Table 2. Overall, the dimensions of the 3D-printed specimen matched well with the designed one. According to the data shown in Table 2, the length, width and thickness of the proposed 3D printed specimens were about 89.90 ± 0.329, 48.49 ± 0.175 and 10.07 ± 0.101 mm, respectively. The main dimension fluctuations occurred at the measuring points located near the fringing areas of the 3D printed specimens. Further, the surface roughness (*Ra*) of the 3D printed specimens after post-processing was about 16 μm. The slight dimensional fluctuation of the 3D printed specimens was due to the distortion induced by the residual stress and the stretching of the supporting structures. During the 3D printing process, materials were stacked by layers. At the same time, internal stress was accumulated within the structure. However, the supporting structures limited the release of the internal stress during manufacturing, and the heating process in the post-treatment minimized the internal stress partly. The internal stress could not be eliminated completely, leading to the geometry dimension changes and structural distortion.

Uniaxial tension and compression tests were conducted to evaluate the Poisson’s ratios of the 3D-printed metamaterial specimen. A small amount of structural distortion due to the viscoelasticity of the 3D printing material is neglected. In the tensile tests, a high accuracy desktop-level testing system, eXpert 5951 (ADMET, Norwood, MA, USA), was used. Before loading, the specimens were fixed by a pair of grips, of which the bottom one was fixed on the platform while the top one was loaded through a load cell (1010FGR-1K-B) with a measuring range of 1000 lbs. The alignment of the two grips was carefully adjusted to avoid any initial distortion to the specimen. During a test, the loading was applied in the displacement-controlled mode, gradually from 0 to 0.09 strain, with an incremental step of 0.005 strain. To calculate the Poisson’s ratios, the overall deformation of the metamaterial was recorded by a high-resolution camera (Canon 80D single-lens reflex camera).

A universal testing system, INSTRON 3369 (INSTRON, Norwood, MA, USA), was used for conducting the uniaxial compressive tests. This system could provide high load accuracy and data acquisition. Apart from the displacement sensor in the load-cell (INSTRON-52154), which could monitor the longitudinal deformation, the transverse deformation of metamaterial was recorded by a high-resolution camera. The specimen was loaded in a stepwise manner, with an increment of 0.0025 strain and holding time of 5s for imaging. After the tests, a grid layer was placed on the original photos by Photoshop software to assess the transverse deformations accurately. Further, static tensile tests have conducted to measure its structural strength and tensile modulus by following the ASTM D638 standard [35]. During this test, a displacement-controlled load was applied to the specimen with a speed of 5 mm/min. Three specimens were tested for each condition. Sensors in the load cell recorded the developments of stress and displacement loads until failure.

## 3. Results

### 3.1. Finite Element Analysis Results

Poisson’s ratio can calculate the ratio of transverse strain and longitudinal strain in elastic loadings, as shown in the following equation [8,40]:

(1) $$v=-\frac{\varepsilon_{1}}{\epsilon }$$

where $\varepsilon_{1}$ is the transverse strain and ϵ is the longitudinal strain.

Poisson’s ratio reflects the lateral deformation of the material as an elastic constant. Transverse strain and longitudinal strain are directional vectors. For conventional engineering materials, the transverse strain and longitudinal strain are usually opposite under unidirectional load. By adjusting with a negative sign, the Poisson’s ratios of such materials are positive. For the NPR metamaterial, however, the ratio of transverse strain and longitudinal strain is positive. The introduction of the negative sign shows the dilational effects of materials. The average lateral displacement of the side panels on this 2D CS-based metamaterial structure was used to calculate the transverse strain in the experiment. Combining the longitudinal strain recorded by the sensors in the load cell, the experimental Poisson’s ratios are obtained. The definition of Poisson’s ratio applies to the elastic deformation region of a material. According to the pre-test result, it was clear that the elastic deformation region of this material was up to the strain of 0.15. Therefore, the corresponding transverse strain should be lower than 0.15. Accordingly, the displacement controlled loads for the tensile and compressive tests were set as the strain of 0.09 and 0.03, respectively.

**Figure 4:**
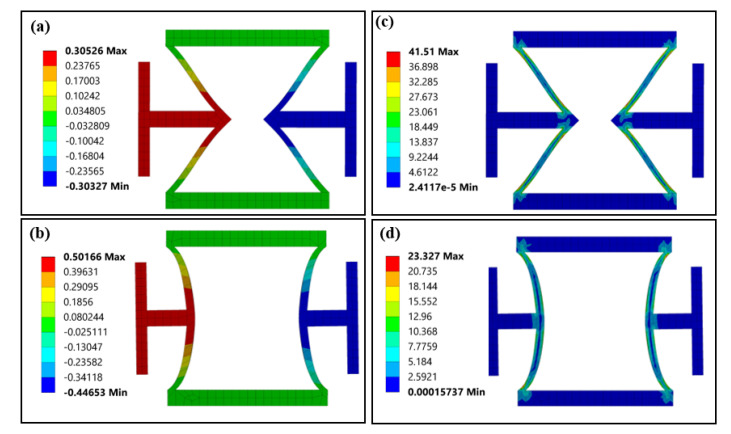
The FEA results of the re-entrant and CS-based units under uniaxial tension (strain 0.05): (**a**) transverse displacement (unit: mm) of the re-entrant unit, (**b**) transverse displacement (unit: mm) of the CS-based unit, (**c**) internal stress distribution (Von Mises equilibrium stress) of the re-entrant unit (unit: MPa) and (**d**) internal stress distribution (Von Mises equilibrium stress) of the CS-based units (unit: MPa).

Figure 4a,b compare the transverse displacement distribution of the conventional re-entrant unit and CS-based unit. The middle connection beams in both units displace outward (expand) under tensile loadings, indicating a negative Poisson’s ratio. Moreover, the transverse displacement in the CS-based unit is more significant than that in the re-entrant unit. As a result, the calculated Poisson’s ratio of the CS-based unit (−1.580) gets a 55.80% increase compared to that of the re-entrant unit (−1.014). Figure 4c,d shows the Von Mises equilibrium stress distributions of two cases. The maximum stress in the re-entrant unit is 41.51 MPa, which is 44.02% higher than that (23.237 MPa) of the CS-based unit. High-pressure areas are mainly concentrated at the joint part connecting cylindrical-shell beams and straight connection beams. These results confirmed the superiority of CS-based unit in increasing the structural negative Poisson’s ratio and reducing the internal stress.

**Figure 5:**
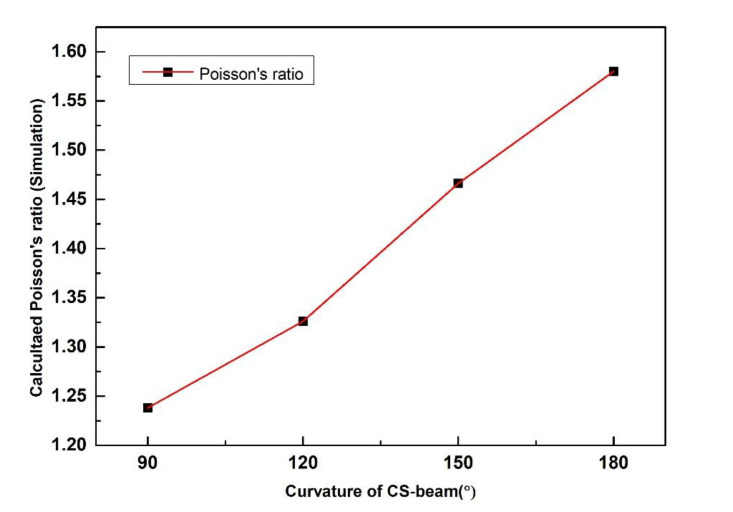
Calculated Poisson’s ratio (absolute value) of CS-based units with different CS-beam curvature based on simulation.

Figure 5 shows the calculated Poisson’s ratio (absolute value) of CS-based units as a function of the curvature of the cylindrical-shell beam with a constant strain of 0.05. Although units with different cylindrical shell curvatures demonstrate the transverse expansion similarly under the uniaxial tensile loadings, the maximum displacements and correspond Poisson’s ratio were quite different. The calculated Poisson’s ratios are −1.238, −1.326, −1.466 and −1.580 for the four cases (90°, 120°, 150° and 180°), respectively. Simulation results confirmed that the Poisson’s ratio almost increased linearly with the curvature of the cylindrical shells.

**Figure 6:**
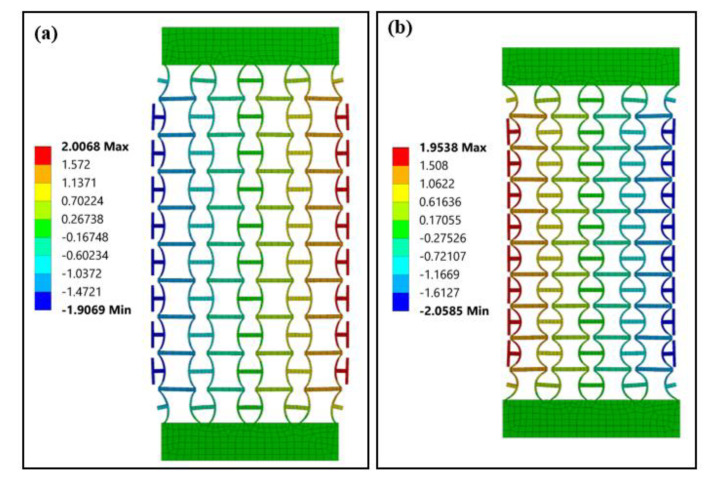
Transverse displacement (unit: mm) distributions of the structure model under (**a**) tension and (**b**) compression.

Figure 6a,b show the transverse displacement distribution of the structural model built by 180° CS-based units under tensile and compressive loadings (0.05 strain), respectively. The structure demonstrates uniform expansion under tensile loadings and shrinkage under compressive loadings, which is a typical negative Poisson’s ratio behavior. The distortion in the top and bottom layers are due to the constraint from the two end blocks. The calculated Poisson’s ratios for the two cases are −1.618 and −1.657, respectively. The introduction of side panels results in the differences in Poisson’s ratio of CS-based units and the structural model. According to the literature [41], for the NPR metamaterials, the structural expansion or shrinkage accompanied by the distortion of units near loading end and boundaries. As shown in Figure 6a, the distortion of top layer units is limited by the top panel at one end. However, it pushes the neighboring units in the second layer for further expansion at the other end. Similar distortion happened to the bottom layer units. As a result, these distortions enhanced the further structural expansion of metamaterials, and the corresponding structural Poisson’s ratio is slightly larger than it of units.

### 3.2. Experimental Test Results

**Figure 7:**
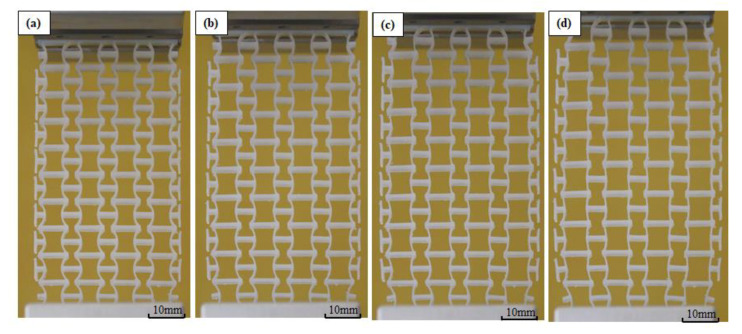
Deformed specimen under tensile loadings: (**a**) initial state, (**b**) strain 0.03, (**c**) strain 0.06 and (**d**) strain 0.09.

**Figure 8:**
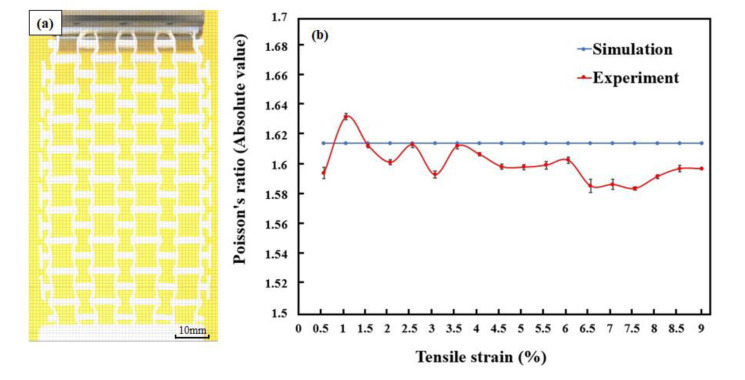
(**a**) Processed image of specimens under tension (strain = 0.09) and (**b**) the changes in Poisson’s ratios.

Figure 7 shows the recorded images of the 3D-printed specimen under uniaxial tensile loads. The specimen was deformed gradually up to the longitudinal strain of 0.05 and further to 0.09, and continued to expand transversely during the tension process. To calculate the Poisson’s ratio, the images were further processed using graphics software. Particularly, digital grid layers were added onto the original images (Figure 8a), and these layers would be used as references for the Poisson’s ratio calculation. Furthermore, according to the FEA simulation results, the distortions of units at the top and bottom layer lead to significant local effects. To improve the measurement accuracy, only the 4 × 8 unit array in the middle is used in calculating structural Poisson’s ratio.

Figure 8b shows the fluctuations in Poisson’s ratios with the increased tensile strain. For comparison, the Poisson’s ratios predicted by numerical simulations were also added into the figure. The numerical Poisson’s ratio is constant (−1.618) while the experimental Poisson’s ratio fluctuates. The numerical simulation results reflected the transverse deformation response under a uniaxial load with ideal quality of specimens and loading environment. In the FEA simulation, the structural deformation was the integration of the deformation of nodes. During this process, the mutation in structural deformation due to the defects in the manufacturing process of the specimens and uneven distribution of loads in actual experiments are neglected. Therefore, the simulated Poisson’s ratio appeared with a straight line for both tensile and compressive loading conditions. However, due to the influence of specimen quality and actual experimental environment, the experimental Poisson’s ratios are fluctuated and slightly lower than that of simulation results after the strain of 0.01. The experimental Poisson’s ratio is −1.598 at the strain of 0.05 and −1.602 at the strain of 0.09. It was identified that the slight differences between the experimental and simulation results are because of the manufacturing defects. In the process of 3D printing, it was almost impossible to remove the supporting material completely without influencing the objects. To maintain the structural integrity of the objects and avoid surface damages, a small amount of the supporting material near the joints has remained. These materials have negatively affected the structural explanation, particularly at the initial deformation stage (0–0.01 strain), leading to the significant fluctuation of Poisson’s ratio.

**Figure 9:**
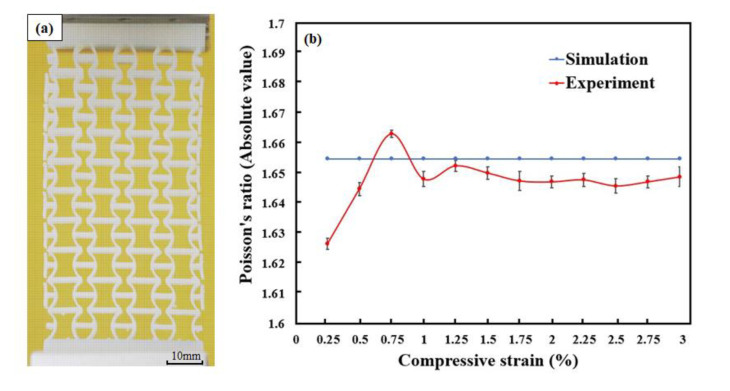
(**a**) Processed image of specimens under compression (strain = 0.03) and (**b**) the changes in Poisson’s ratios.

Figure 9a shows a processed image under compression at the strain of 0.03 with a grid layer for calculating the experimental Poisson’s ratio. Figure 9b compares the Poisson’s ratios obtained by the experiment and simulation. Similar to the results in tension tests, the predicted Poisson’s ratio is a constant value (−1.657). In the experiment, however, the manufacture defects led to the fluctuation of the Poisson’s ratio, particularly in the initial stage. The experimental Poisson’s ratio is −1.652 at the strain of 0.03. As the experiment progresses, it was found that a significant out-plane distortion occurred after the longitudinal strain was over 0.03. With the out-plane distortion developed, the recorded transverse shrinkage of 2D specimens was no longer the main deformation mode. Therefore, the deformation of 0–0.03 longitudinal strain was analyzed only.

**Figure 10:**
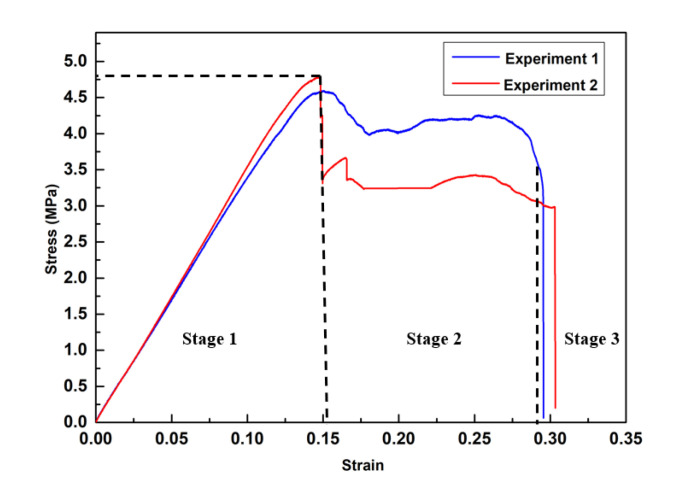
Experimental stress–strain curves of CS-based metamaterial units.

Figure 10 presents the stress–strain curve of CS-based metamaterial units. This curve follows three stages. In stage 1, the stress–strain curves showed an almost linear increment and reached their maximum stress value. In this stage, the elastic deformation of the internal structure was the principal deformation mode. A drop in stress occurred at the end of stage 1. In stage 2, the plastic deformation occurred and gradually replaced the elastic deformation as the main deformation mode. In this stage, specimens suffered internal structure damage due to the joint fracture and separation between the CS-beams and the thin connections. These damages originated from one unit, and developed to others. Therefore, it is reasonable to observe a sharp stress drop from 4.78 to 3.85 MPa in Experiment 2. When internal damage accumulated to a certain degree, the overall structure was destroyed, as shown in stage 3.

The average calculated ultimate tensile strength based on tests is 4.78 MPa, while the tensile modulus of the elastic region is about 57.7 MPa. The mechanical properties, especially tensile strength and modulus of a unit cell, depend on the raw material and processing parameters. The experimental results indicated the mechanical properties of durable resin under the abovementioned processing and testing conditions. The tensile strength and modulus can be improved by changing the material or optimizing the structure and post-processing conditions.

## 4. Discussion

### 4.1. Universality of Cylindrical-Shell-Based Structures

**Figure 11:**
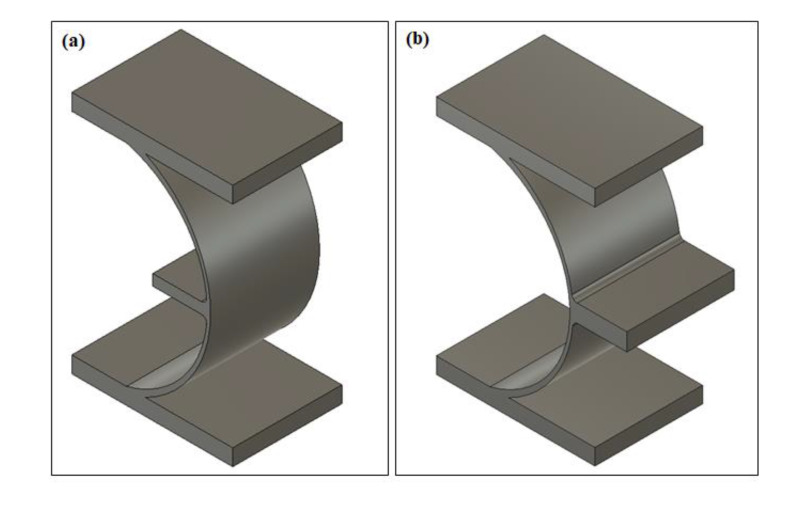
(**a**) Negative element, (**b**) positive element.

The deformation mechanism of proposed CS-based NPR metamaterial can be explained using a fundamental element, as shown in Figure 11a. This element consists of horizontal beams and one 180° cylindrical shell. The horizontal beams can keep the whole structure connected, and the cylindrical shell carries the major deformation. Similarly, one can also develop another type of element (Figure 11b), which can deform oppositely. For simplicity, we named the former element as the negative element (NE) and the latter as the positive element (PE). Then new metamaterials with tailorable Poisson’s ratio could be designed by combining these two types of elements. For example, two NEs could form a negative Poisson’s ratio unit (Figure 1a), while two PEs could form a positive Poisson’s ratio unit. With the combination of one PE and one NE, a zero Poisson’s ratio unit would be created. The transverse deformations of these two elements are cancelled in an individual unit, leading to a constant transverse dimension and thus a zero Poisson’s ratio. These rules can also be applied to explain the deformation mechanisms of most existing paper-folding-inspired metamaterials [5,6,22,23,31,32,42,43,44,45,46].

### 4.2. Wearable Generator Based on Cylindrical-Shell-Based NPR Metamaterial

Our new NPR metamaterial has an advanced deformation mode under uniaxial loadings, which makes it an ideal material for wearable generators. An energy harvesting integrated insole using piezoelectric ceramic could generate an intermittent signal by stretching piezoelectric ceramic beams [47]. However, the piezoelectric ceramic in their energy harvesting unit was expanded when the insole was compressed. Therefore, gaps were necessary between adjacent piezoelectric ceramic units. This reduced the number of units in the harvester with a fixed volume and further decreased power output. With the generator unit using piezoelectric films and our new NPR metamaterial, the transverse dimension will reduce under compression. As a result, gaps between adjacent units could be minimized or even cancelled, leading to an increase in power density by a higher space utilization. In a new design, the electricity generated by piezoelectric material will be collected and stored by external circuits. This CS-based NPR metamaterial generator could continuously provide power to other wearable devices without an extra battery, showing a great prospect in the future.

## 5. Conclusions

This work has successfully developed a new mechanical metamaterial based on a cylindrical-shell-based unit. The properties of this new metamaterial have been thoroughly explored by the finite element simulation and experimental verification. Compared to the conventional re-entrant metamaterial, the new metamaterial possesses a larger Poisson’s ratio (55.80% increase) and a lower stress concentration (44.02% reduction). Given the universality of the proposed CS-based structure, one can easily develop more metamaterials with tailorable Poisson’s ratios. A wearable generator based on CS-based units shows its prospect in the wearable device field.

## Figures and Tables

**Table 1:** Various types of Poisson’s ratio related metamaterials and their limitations.

Name	Advantage	Limitation
Star-shaped-pore metamaterials [10]	Large negative Poisson’s ratio (Poisson’s ratio: −1)	Soft substrate materials, flat structure.
Bistable bridge metamaterials [16]	Unlimited extension with almost zero Poisson’s ratio	Introduce impulse to the structure.
Kirigami cellulars [21]	Customized Poisson’s ratio with simple internal structures	Stress concentration.
Paper-folding inspired metamaterials [22]	Customized Poisson’s ratio with a continues deformation	Poor structural stability with thin beams.

**Table 2:** The size of 3D-printed specimens.

	Length (mm)	Width (mm)	Thickness (mm)
Maximum measured value	90.54	48.73	10.30
Minimum measured value	89.50	48.20	9.95
Average measured value	89.90	48.49	10.07
Standard deviation	0.329	0.175	0.101
Sizes of the digital model	90.00	48.40	10.00

## References

[1] Yu X., Zhou J., Liang H., Jiang Z., Wu L. (2018). Mechanical metamaterials associated with stiffness, rigidity and compressibility: A brief review. Prog. Mater. Sci..

[2] Ren C., Yang D., Qin H. (2018). Mechanical performance of multidirectional buckling-based ngative stiffness metamaterials: An analytical and numerical study. Materials..

[3] Zadpoor A.A. (2016). Mechanical meta-materials. Mater. Horiz..

[4] Miller W., Smith C.W., Mackenzie D.S., Evans K.E. (2009). Negative thermal expansion: A review. J. Mater. Sci..

[5] Li S., Hassanin H., Attallah M.M., Adkins N.J.E., Essa K. (2016). The development of TiNi-based negative Poisson’s ratio structure using selective laser melting. Acta Mater..

[6] Schwerdtfeger J., Heinl P., Singer R.F., Körner C. (2010). Auxetic cellular structures through selective electron-beam melting. Phys. Status Solidi B Basic Res..

[7] Bose P., Pandey K.M. (2012). Desirability and Assessment of Mechanical Strength Characteristics of Solid Propellant for Use in Multi Barrel Rocket Launcher. Int. J Chem. Eng..

[8] Zhang L. (2001). Solid Mechanics for Engineers.

[9] Lakes R.J. (1987). Foam structures with a negative Poisson’s ratio. Science.

[10] Mizzi L., Mahdi E.M., Titov K., Gatt R., Attard D., Evans K.E., Grima J.N., Tan J.C. (2018). Mechanical metamaterials with star-shaped pores exhibiting negative and zero Poisson’s ratio. Mater. Des..

[11] Bacigalupo A., Gambarotta L. (2014). Homogenization of periodic hexa- and tetrachiral cellular solids. Compos. Struct..

[12] Alderson A., Alderson K.L., Attatd D., Evans K.E., Gatt R., Grima J.N., Miller W., Ravirala N., Smith C.W., Zied K. (2010). Elastic constants of 3-, 4- and 6-connected chiral and anti-chiral honeycombs subject to uniaxial in-plane loading. Compos. Sci. Technol..

[13] Nežerka V., Somr M., Vorel J., Doskar M., Antos J., Zeman J., Novak J. (2018). A jigsaw puzzle metamaterial concept. Compos. Struct..

[14] Taylor M., Francesconi L., Gerendas M., Shanian A., Carson C., Bertoldi K. (2014). Low porosity metallic periodic structures with negative Poisson’s ratio. Adv. Mater..

[15] Hewage T., Alderson K., Alderson A., Scarpa F. (2016). Double—Negative Mechanical Metamaterials Displaying Simultaneous Negative Stiffness and Negative Poisson’s Ratio Properties. Adv. Mater..

[16] Yang H., Ma L. (2018). Multi-stable mechanical metamaterials with shape-reconfiguration and zero Poisson’s ratio. Mater. Des..

[17] Qiu J., Lang J.H., Slocum A.H. (2004). A Curved-Beam Bistable Mechanism. J. Microelectromech. Syst..

[18] Larsen U.D., Signund S., Bouwsta S. (1997). Design and fabrication of compliant micromechanisms and structures with negative Poisson’s ratio. J. Microelectromech. Syst..

[19] Rafsanjani A., Akbarzadeh A., Pasini D. (2015). Snapping mechanical metamaterials under tension. Adv. Mater..

[20] Shan S., Kang S.H., Raney J.R., Wang P., Fang L., Candido F., Lewis J.A., Bertoldi K. (2015). Multistable Architected Materials for Trapping Elastic Strain Energy. Adv. Mater..

[21] Neville R., Scarpa F., Pirrera A. (2016). Shape morphing Kirigami mechanical metamaterials. Sci. Rep..

[22] Olympio K.R., Gandhi F. (2009). Zero Poisson’s Ratio Cellular Honeycombs for Flex Skins Undergoing One-Dimensional Morphing. J. Intell. Mater. Syst..

[23] Broccolo S.D., Laurenzi S., Scarpa F. (2017). AUXHEX – A Kirigami inspired zero Poisson’s ratio cellular structure. Compos. Struct..

[24] Evans K., Kansah M., Hutchinson I. (1992). Modelling negative Poisson ratio effects in network-embedded composites. Acta Mater..

[25] Li D., Yin J., Dong L., Lakes R. (2018). Strong re-entrant cellular structures with negative Poisson’s ratio. J. Mater. Sci..

[26] Fu M., Chen Y., Hu L. (2017). A novel auxetic honeycomb with enhanced in-plane stiffness and buckling strength. Compo. Struct..

[27] Li D., Gao R., Dong L., Lam W., Zhang F. (2019). A novel 3D re-entrant unit cell structure with negative Poisson’s ratio and tunable stiffness. Smart Mater. Struct..

[28] Jiang W., Ma H., Wang J., Feng M., Qu S. (2016). Mechanical metamaterial with negative Poisson’s ratio based on circular honeycomb core. Chin. Sci. Bull..

[29] Yang H., Ma L. (2019). Multi-stable mechanical metamaterials by elastic buckling instability. J. Mater. Sci..

[30] Liu J., Tang J., Ahmad R., Ma Y. (2019). Meta-Material Topology Optimization with Geometric Control. Comput. Aided Des. Appl..

[31] Lira C., Scarpa F., Tai Y.H., Yates J.R. (2011). Transverse shear modulus of SILICOMB cellular structures. Comps. Sci. Technol..

[32] Chen Y., Scarpa F., Remillat C., Farrow I., Liu Y., Leng J. (2013). Curved Kirigami SILICOMB cellular structures with zero Poisson’s ratio for large deformations and morphing. J. Intell. Mater. Syst. Struct..

[33] Durable Resin Technical. https://formlabs-media.formlabs.com/datasheets/1801084-TDS-ENUS-0P.pdf.

[34] Xu T., Yoo J.H., Babu S., Roy S., Lee J.B., Lu H. (2016). Characterization of the mechanical behaviour of SU-8 at microscale by viscoelastic analysis. J. Micromech. Microeng..

[35] ASTM (2014). D638-14 Standard Test Methods for Tensile Properties of Plastics.

[36] ASTM (2015). D695-15 Standard Test Method for Compressive Properties of Rigid Plastics.

[37] Zuo Z., Gong J., Huang Y., Zhan Y., Gong M., Zhang L. (2019). Experimental research on transition from scale 3D printing to full-size printing in construction. Constr. Build Mater..

[38] Weng Z., Zhou Y., Lin W., Senthil T., Wu L. (2016). Structure-property relationship of nano enhanced stereolithography resin for desktop SLA 3D printer. Compos. Part A Appl. Sci. Manuf..

[39] Borrello J., Nasser P., Iatridis J.C., Costa K.D. (2018). 3D printing a mechanically-tunable acrylate resin on a commercial DLP-SLA printer. Addit. Manuf..

[40] Hou X., Silberschmidt V. (2015). Metamaterials with Negative Poisson’s Ratio: A Review of Mechanical Properties and Deformation Mechanisms. Mech. Adv. Mater..

[41] Ling B., Wei K., Wang Z., Yang X., Qu Z., Fang D. (2020). Experimentally program large magnitude of Poisson’s ratio in additively manufactured mechanical metamaterials. Int. J. Mech. Sci..

[42] Wang F. (2018). Systematic design of 3D auxetic lattice materials with programmable Poisson’s ratio for finite strains. J. Mech. Phys. Solids.

[43] Huang J., Zhang Q., Scarpa F., Liu Y., Leng J. (2016). Bending and benchmark of zero Poisson’s ratio cellular structures. Compos Struct.

[44] Levy O., Krylov S., Goldfarb I. (2006). Design considerations for negative Poisson ratio structures under large deflection for MEMS applications. Smart Mater. Struct..

[45] Wang X., Li X., Ma L. (2016). Interlocking assembled 3D auxetic cellular structures. Mater. Des..

[46] Xiong J., Gu D., Chen H., Dai D., Shi Q. (2017). Structural optimization of re-entrant negative Poisson’s ratio structure fabricated by selective laser melting. Mater. Des..

[47] Turkmen A.C., Celik C. (2018). Energy harvesting with the piezoelectric material integrated shoe. Energy.

